# Underwater Acoustic Orthogonal Frequency-Division Multiplexing Communication Using Deep Neural Network-Based Receiver: River Trial Results

**DOI:** 10.3390/s24185995

**Published:** 2024-09-15

**Authors:** Sabna Thenginthody Hassan, Peng Chen, Yue Rong, Kit Yan Chan

**Affiliations:** School of Electrical Engineering, Computing and Mathematical Sciences (EECMS), Faculty of Science and Engineering, Curtin University, Bentley, WA 6102, Australia; sabna.thengint@postgrad.curtin.edu.au (S.T.H.); peng.chen@curtin.edu.au (P.C.); kit.chan@curtin.edu.au (K.Y.C.)

**Keywords:** underwater acoustic communication, orthogonal frequency-division multiplexing, long short-term memory, deep neural network, convolutional neural network, Doppler shift, least squares

## Abstract

In this article, a deep neural network (DNN)-based underwater acoustic (UA) communication receiver is proposed. Conventional orthogonal frequency-division multiplexing (OFDM) receivers perform channel estimation using linear interpolation. However, due to the significant delay spread in multipath UA channels, the frequency response often exhibits strong non-linearity between pilot subcarriers. Since the channel delay profile is generally unknown, this non-linearity cannot be modeled precisely. A neural network (NN)-based receiver effectively tackles this challenge by learning and compensating for the non-linearity through NN training. The performance of the DNN-based UA communication receiver was tested recently in river trials in Western Australia. The results obtained from the trials prove that the DNN-based receiver performs better than the conventional least-squares (LS) estimator-based receiver. This paper suggests that UA communication using DNN receivers holds great potential for revolutionizing underwater communication systems, enabling higher data rates, improved reliability, and enhanced adaptability to changing underwater conditions.

## 1. Introduction

Underwater communication can be performed using radio frequency (RF) signals, optical signals, or acoustic signals [1]. RF signals can penetrate water to a certain extent and offer potential for short-range communication or underwater localization. These signals are heavily attenuated in water, especially at higher frequencies, and are only efficient in the extra-low-frequency range (30–300 Hz). The range of RF communication is limited, and signal quality is likely to be affected by the conductivity and salinity of the water. Optical communication uses light waves to transmit data through water and offers high data rates and low latency. These signals are highly attenuated in water, especially in turbid or murky conditions. Light scattering and absorption by particles, plankton, and dissolved substances in the water further limit the range (less than 100 m) and reliability of optical communication [1]. Acoustic communication is the most commonly used method for underwater communication. It involves transmitting and receiving sound waves through water. Although this method provides low data rates, acoustic waves can travel long distances underwater and penetrate through obstacles and sediment, making them suitable for long-range communication [1].

The importance of underwater acoustic (UA) communication is drastically changed in recent years as its applications have begun to shift from military toward commercial [2]. High-data-rate UA communication is possible using orthogonal frequency-division multiplexing (OFDM) with its strong capability in mitigating inter-symbol interference (ISI) with a large delay spread [2]. Communicating reliably through a UA channel is inherently difficult because of its limited bandwidth, extensive multipath interference, and fast time variation resulting from Doppler shifts. In a multipath channel, UA communication results in the arrival of signals from various paths [2]. OFDM communication is used to alleviate the impacts of this multipath interference [3].

Channel estimation is the key element in a communication system. Linear interpolation is used for channel estimation in conventional methods [3]. However, strong non-linearity in UA channels, due to the large delay spread of the multipath channel, cannot be modeled precisely as the channel delay profile is unknown in practice. In contrast to traditional methods like least squares (LS), which typically rely on linear relationships between variables, a deep neural network (DNN) can model complex, non-linear relationships. This ability allows it to detect intricate patterns in data that linear models may fail to capture. The neural network (NN)-based receiver is effective in learning non-linearity through NN training [4].

In recent years, machine learning has become a powerful approach for modeling and pattern recognition in complex systems. Applications include emotion recognition using brain-activity sensors [5], vehicle type classification [6], and speech separation techniques [7]. An NN is composed of multiple layers, each with a set of neurons that perform a weighted sum of inputs, followed by a non-linear activation function, with the output then passed to the next layer [8]. During training, the weights and biases are fine-tuned based on the provided data. In supervised deep learning (DL), a loss function guides this process, optimizing the weights and biases to improve the accuracy of the model [9]. An OFDM-based receiver has been developed by integrating an NN for UA communication before and proved that non-linearity in UA channels can be compensated for using an NN [10] where the number of bits recovered is low when Doppler is introduced.

This paper presents a DL-based UA OFDM communication system. In contrast to conventional UA OFDM communication system receivers that rely on channel estimation and equalization to detect transmitted symbols, the neural network receiver is able to recover them from received data through proper training. River trials are conducted in the Canning River, Western Australia in summer to investigate the performances of three NN-based receivers: a multi-layer perceptron (MLP) network, a convolutional neural network (CNN), and a long short-term memory (LSTM) network. These are the three architectures that are basic, most popular, and suitable for sequential problems, and it is worth comparing their performance in the same scenario. The results show that the NN-based receiver outperforms the conventional LS estimator-based receiver.

The remainder of this paper is as follows. In Section 2, previous research works on conventional UA OFDM communication systems and NN-based UA OFDM communication systems are presented. The background information regarding each component in the system is explained in Section 3. The proposed system design is explained in Section 4, which includes an overview of the architecture of the proposed network and the training process. In Section 5, the experimental setup is detailed. The performance results of the proposed receiver are presented in detail in Section 6. Finally, conclusions and future works are drawn in Section 7.

## 2. Literature Review

UA communication is challenging yet crucial for various applications such as ocean exploration, underwater navigation, and environmental monitoring [11]. Over the years conventional UA OFDM systems have been modified to increase the accuracy and performance of UA OFDM receivers.

### 2.1. Review of Conventional UA OFDM Communication Systems

A real-time OFDM-based adaptive UA communication system is proposed in [11]. The received signal-to-noise ratio (SNR) serves as a performance metric for determining transmission parameters, which are then fed back to the transmitter for data transmission. This paper examines how adaptive modulation schemes in a non-stationary UA environment allow for the dynamic selection of subcarriers, modulation sizes, and power allocation, thereby improving communication reliability. This strategy ensures continuous connectivity and increases data rates. In [12], two algorithms are proposed for joint channel estimation and impulsive noise mitigation in UA OFDM systems. These algorithms use pilot subcarriers to estimate impulsive noise and channel impulse response. These new algorithms improve the accuracy of channel estimation and the effectiveness of impulsive noise mitigation by utilizing the natural structure of OFDM signals, in contrast to the current blanking methods. The authors of [13] suggest an altered OFDM system utilizing fast Walsh–Hadamard transform (FWHT) combined with low density parity check (LDPC) coding. By utilizing Zadoff–Chu (ZC) sequences for time synchronization, the suggested algorithm can eliminate the time offset linked to the FWHT. A survey of the technical issues, challenges and future directions in underwater environmental communication are provided in [14]. This study offers a comprehensive evaluation of modern UA methods, suggesting possible paths for development and recommendations to improve future wireless networking systems in underwater settings. Furthermore, the main efforts and impacts of the present wireless communication approaches in the underwater environment, with a focus on improving service quality and energy efficiency over extended distances, are emphasized.

### 2.2. Review of DL-Based UA Communications

In 2023, ref. [15] introduced a downlink non-orthogonal multiple access (NOMA) UA communication system using a DNN with a one-dimensional CNN. The proposed system is evaluated in two scenarios. In the first scenario, two users with different power levels and distances from the transmitter utilize binary phase-shift keying (BPSK) and quadrature phase-shift keying (QPSK) modulations to enable multi-user communication. In the second scenario, all three users employ BPSK modulation. For training the model, a composite signal is transmitted through samples of the UA channel and provided to the model along with labels. The DNN receiver learns the characteristics of the UA channel independently of channel state information (CSI). The performance is compared to the traditional successive interference cancellation (SIC) receiver, with simulation results showing that the DNN-based DL NOMA UA receiver outperforms the SIC receiver in terms of bit error rate (BER) across all modulation orders. In 2022, a design was proposed in [16] using DL-based signal detection for UA orthogonal time frequency space (OTFS) communication. Unlike the proposed DNN-based UA OFDM communication, ref. [15] used NOMA as the multi-access technique and [16] used OTFS as a modulation scheme instead of OFDM. A hybrid NN was used in [17] for modulation recognition in UA communication. In this study, an NN model named R&CNN was developed for the effective and accurate identification of modulation types in underwater acoustic signals. The model was designed to leverage the strengths of both recurrent neural networks (RNNs) and CNNs in processing underwater acoustic signal data. Moreover, the proposed model was involved with fewer parameters and lower time complexity; the proposed method was effective for real-time communication systems. Ref. [18] examined how DL and various traditional machine learning techniques can effectively learn and model the UA channel using real data obtained from a water tank with disturbances and from a lake. This method incorporated DNNs and LSTM networks to model the underwater acoustic channel. The experimental results indicated that DL offered an enhanced performance for channel modeling compared to traditional machine learning techniques, as reflected in a lower mean absolute percentage error. Even though [17,18] utilized DNN architecture in UA communication, the purpose of these studies was different from our proposed method. The method in [17] was used for identifying modulation schemes and the method in [18] was used for channel modeling.

### 2.3. Review of DL-Based Receiver for UA OFDM Communications

In 2022, refs. [19,20] introduced skip connections in a DNN estimator with fully connected layers and in a CNN estimator that incorporated both CNN layers and fully connected layers, respectively. The original transmitted symbols were reconstructed from the received signals by effectively extracting promising features from the received signals with the stacks of fully connected layers in [19]. In [20], the convolutional layers with skip connections were used for feature extractions, while demodulation was performed by an MLP after that. The purpose of these two papers was to perform signal recovery in UA OFDM communication similarly to the proposed DNN-based UA OFDM communication. However, the complexity of the architecture in both papers was high when compared to the proposed design. In 2019 [10], an OFDM-based receiver was developed by integrating an NN for UA communication which considered the complicated UA communication system as a DNN. In comparison to conventional UA communication, DL-based UA communication uses training to understand the complex disruptions caused by the UA channel and subsequently decodes the transmitted symbols directly from the received signal. The DL-based UA OFDM communication system was trained and tested utilizing a ray tracing toolbox with a sound speed profile (SSP) obtained from a real sea experiment. The architecture of the designed DNN was similar to the proposed MLP network, but the parameters were different. The performance of the approach in [10] was poor in terms of recovered bits when a Doppler shift existed in the system.

## 3. System Model

A frame-based UA OFDM communication system [21] is considered in this research. Every OFDM frame is divided into a pilot block and a data block, where the pilot block assists in gathering CSI during the channel estimation. A stream of binary bits are converted into symbols from the QPSK constellation in the data block, $d={\left(d\left[1\right],\ldots ,d\left[N_{c}\right]\right)}^{T}$, where $N_{c}$ is the number of data subcarriers. Null subcarriers are introduced at every sixth position in the pilot block, which contains $N_{p}$ pilot subcarriers $p={\left(p\left[1\right],\ldots ,p\left[N_{p}\right]\right)}^{T}$. Inverse fast Fourier transform (IFFT) is applied to each OFDM symbol to convert it into the time domain. To avoid the ISI, a cyclic prefix (CP) with length $T_{cp}$ longer than the channel delay spread is added to the time domain symbol. The received signal can be written as

(1) y(t)=x(t)∗h(t)+w(t)

where x(t) represents the signal being transmitted, ∗ indicates the convolution process, h(t) stands for the impulse response of the channel, and w(t) denotes the additive noise. In order to demonstrate the limitations of the conventional OFDM receiver, let us analyze a multipath UA channel with the frequency response defined at the *k*th subcarrier.

(2) $$H_{k}=\sum_{l=1}^{L}h_{l}e^{-j2\pi f_{k}\tau_{l}},\;k=1,\ldots ,N_{c}$$

where $h_{l}$ and $\tau_{l}$, l=1,…,L, are the amplitude and delay of the *l*th arrival, respectively, $f_{k}=k/T$ is the frequency of the *k*th subcarrier, and *T* is the duration of the OFDM symbol. In traditional OFDM systems with comb-based pilot patterns, pilot subcarriers are utilized for estimating the channel. Linear interpolation is used to determine the channel information on a data subcarrier by estimating the channel coefficients of the two nearest pilot subcarriers. This method is successful in land-based radio systems because the multipath spread is significantly shorter than the length of an OFDM symbol (i.e., $\tau_{l}\ll T$). However, its performance will decrease in UA communication systems, as UA channels normally experience significant delay spread caused by numerous reflections from the sea surface and the seafloor, with later signals possibly having higher amplitudes than earlier ones [22]. In these scenarios, there is a significant non-linear relationship in the channel frequency response (2) between two pilot subcarriers. An NN-based receiver can learn these non-linearities and outperform the conventional method.

Doppler shift compensation in the traditional approach involves minimizing leakage energy in the null subcarriers within the pilot OFDM block [23]. The compensation of carrier frequency offset (CFO) for the received baseband symbol is performed by

(3) $$d\left[n\right]=y\left[n\right]e^{-j2\pi n\hat{f}/B_{w}}$$

where y[n] is the received signal sample, d[n] is the CFO-compensated sample, $\hat{f}$ is the estimated value of the CFO, and $B_{w}$ is the bandwidth. The objective function of the CFO estimation is explained as

(4) $$J\left(f\right)=\sum_{k\in S_{N}}{\left|f_{k}^{H}\Phi^{H}\left(f\right)y\right|}^{2}$$

where $y={\left(y\left[1\right],\ldots ,y\left[N_{c}\right]\right)}^{T}$ is the received data, $S_{N}$ is the set of null subcarriers, and $f_{k}$ and Φ are

(5) $$f_{k}={\left[\;1,e^{j2\pi k/N_{c}},\ldots ,e^{j2\pi k\left(N_{c}-1\right)/N_{c}}\;\right]}^{T}$$

and

(6) $$\Phi =\mathrm{diag}(e^{j2\pi f/B_{w}},\ldots ,e^{j2\pi N_{c}f/B_{w}}).$$

Here, ${\left(.\right)}^{H}$ and diag(.) denote the conjugate transpose and a diagonal matrix, respectively. The estimate of *f* is given by

(7) $$\hat{f}=\arg\min_{f}J(f).$$

### 3.1. Background of DNN

**Figure 1:**
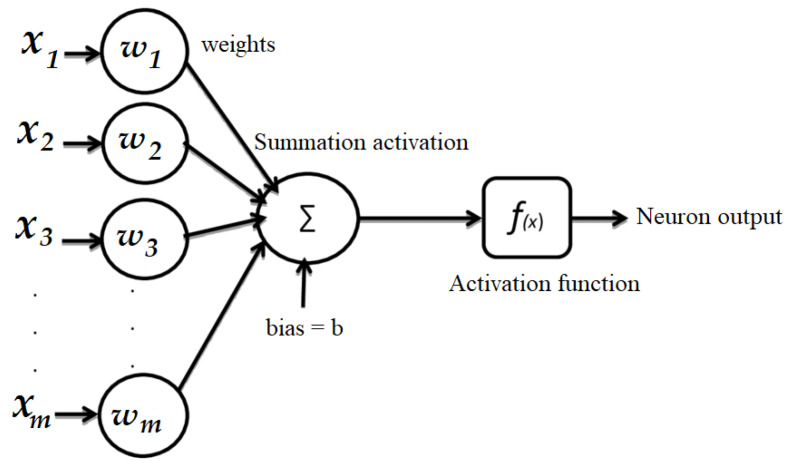
Structure of a neuron.

NNs were developed in the 1940s [24]. After a few years, a mathematical model for an NN was proposed and its unit was a simple formalized neuron [24]. Neurons are the elementary units of an artificial neural network (ANN) as shown in Figure 1.

A neuron is a sum of inputs $x_{1},x_{2},\ldots ,x_{m}$, where *m* is the number of inputs, $w_{1},w_{2},\ldots ,w_{m}$ are the corresponding weights of those inputs and *b* (a numerical value) is the bias value of that neuron:

(8) $$z=\sum_{i=1}^{m}w_{i}x_{i}+b.$$

The output of the neuron can be illustrated as

(9) $$a=f\left(z\right)=f\left(\sum_{i=1}^{m}w_{i}x_{i}+b\right)$$

where f(z) is the activation function [25].

**Figure 2:**
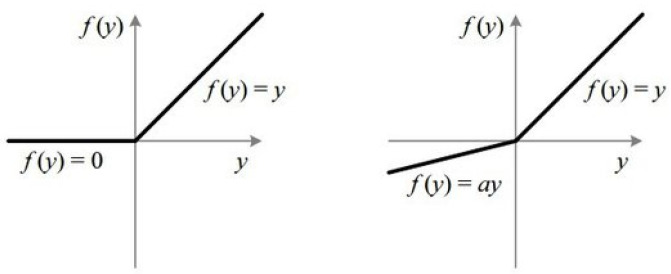
ReLU and leaky ReLU [26].

ReLU is the most commonly used activation function in NNs. Compared to other activation functions such as sigmoid and tanh, ReLU offers a faster convergence rate and calculation speed due to its linear operation. However, ReLU can cause “dead neurons”, which negatively impact an NN’s performance. When the input value is negative, ReLU’s output is always zero, and its first derivative is zero, preventing the neuron from updating its parameters. To address this limitation, Maas introduced a leaky value in the negative half-interval of the ReLU function, known as the Leaky ReLU function as shown in Figure 2 where *a* is a constant [26].

NNs are widely recognized as a powerful modeling tool and are applied across numerous domains, including computer vision, natural language processing, and speech recognition, as well as signal processing tasks such as modulation recognition, signal detection, and channel estimation [10]. DL methods are a type of representation learning that involves multiple levels of representation. These levels are created by composing simple but non-linear modules, each of which transforms the representation at one level (beginning with the raw input) into a representation at a higher, slightly more abstract level. By composing enough of these transformations, very complex functions can be learned [27]. DNNs are composed of an input layer, an output layer, and several hidden layers in between. Each layer consists of input neurons, output neurons, bias, weights, layers, and activation functions that are adjusted according to the training data provided. There are a variety of NNs available such as CNNs and RNNs [28]. DNNs became popular in recent years due to their state-of-the-art capability of the analysis and processing of large amounts of data [28]. A DNN is a multi-layered structure with multiple neurons in a layer. Each neuron has a non-linear activation function (9), which is activated by the inputs and has been adjusted by adaptable weights. Activation in each layer determines the activation in the next layer and adjusts the parameters accordingly [29]. A single-layer NN consists of an input layer and an output layer, which is only applicable for linearly separable functions.

DNNs are capable of learning complex patterns in the data and have been successfully applied to various tasks such as image recognition, speech recognition, and natural language processing [4]. They are “deep” because they have more than one hidden layer, allowing them to learn hierarchical representations of data. The depth and complexity of DNNs enable them to model intricate non-linear relationships and extract high-level features from raw data. Training DNNs involves adjusting the weights and biases of connections between nodes using algorithms such as back-propagation and optimization techniques.

In this paper, three NN architectures—MLP, CNN, and LSTM—and their performances are evaluated. MLP is the most commonly used NN architecture. CNN leverages the ideas of sparse interactions, parameter sharing, and equivariant representations to improve an NN. LSTM plays a crucial role in sequential problems by exploiting its memory blocks [30].

#### 3.1.1. MLP

**Figure 3:**
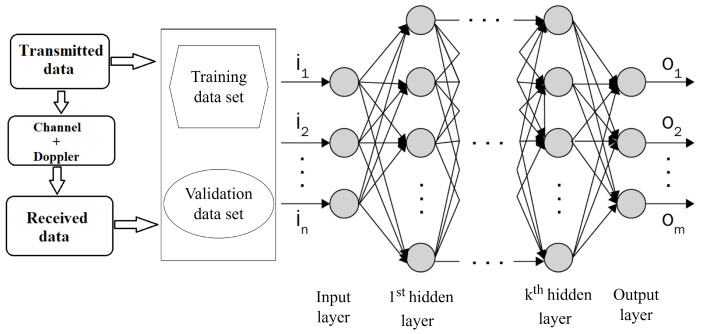
NN training process.

MLP is the most frequently utilized NN consisting of an input layer, an output layer, and a minimum of one hidden layer, as illustrated in Figure 3. It is structured with a forward-feed design since there is no loop for feedback and the output of a neuron does not impact itself. The non-linear activation functions, loss functions, and optimizers play a crucial role in directing the performance of the MLP [31].

#### 3.1.2. LSTM

**Figure 4:**
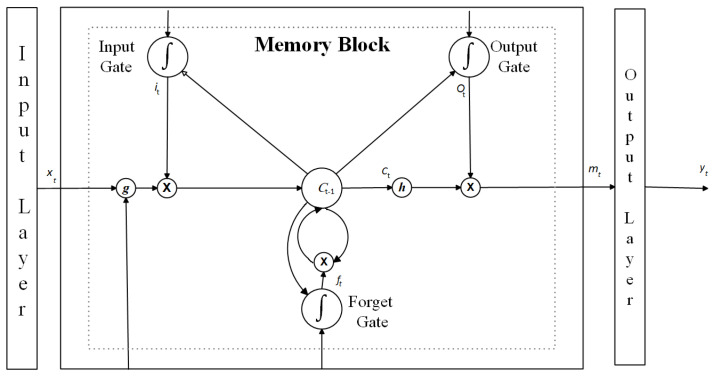
Architecture of an LSTM layer.

For sequential-data-related problems, LSTM is a suitable network to work with. The LSTM network is an extension of RNNs, designed to learn from sequence data [30]. It uses a structure based on short-term memory processes to develop long-term memory. The primary components of an LSTM architecture are the memory block and its regulators, as illustrated in Figure 4. The model input is denoted as $x=(x_{1},x_{2},\ldots ,x_{T})$, and the output sequence is denoted as $y=(y_{1},y_{2},\ldots ,y_{T})$, where *T* is the prediction period. The memory block is depicted within a dashed box and includes an input gate, an output gate, and a forget gate, with their outputs represented as $i_{t}$, $o_{t}$, and $f_{t}$, respectively. The activation vectors for each cell and memory block are denoted as $c_{t}$ and $m_{t}$, respectively, and *g* and *h* are centered logistic sigmoid functions. The cell state serves as the memory unit of the network; storing information can be written to, read from, or preserved from a previous cell state through open and close gates. Information from the previous step also enters the cell state, maintaining relevant data throughout processing. Using an RNN learning process, the gates decide which data to remember and which to discard during training [32]. LSTM can effectively manage time lags exceeding 1000 discrete time steps. This approach utilizes constant error carousels (CECs) to maintain a consistent error flow within specialized cells. Multiplicative gate units learn when to allow access and control entry to these cells. Memory cells in the LSTM layer provide the capability of resetting or retaining the current state of the model and perform additive interactions, which can enhance gradient flow over long sequences during training [33].

#### 3.1.3. CNN

**Figure 5:**
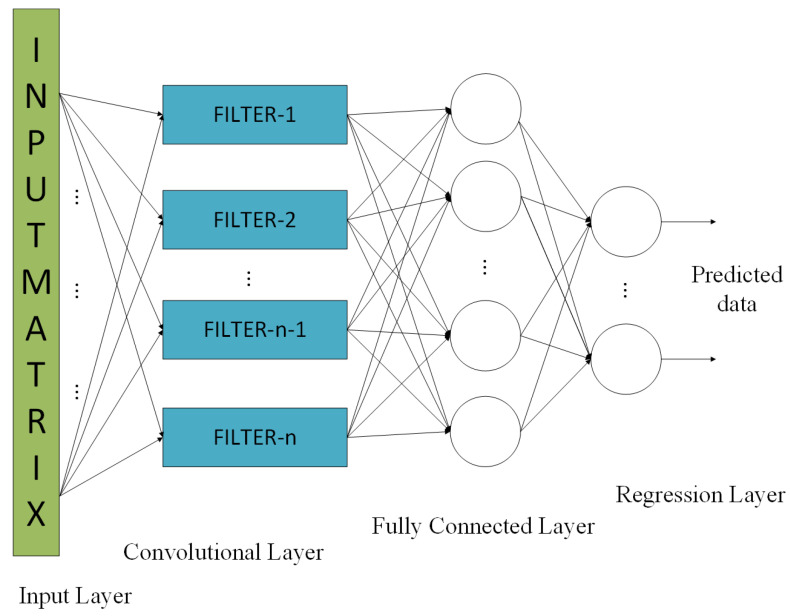
Architecture of the CNN-based receiver.

CNN is a commonly used network for identifying patterns in images, allowing them to recognize objects, classes, and categories [4]. CNN is also highly effective for classifying audio, time-series, and signal data and is composed of several layers, including an input layer, and multiple convolutional layers and fully connected layers as shown in Figure 5. Convolutional layers receive multiple feature maps as input and generate *n* feature maps as output, where *n* represents the number of filters in the convolutional layer. The weights of the filters used in the linear convolutions are the parameters adjusted based on the training data. The filters, also known as kernels, are used to extract the feature compositions of the data given. Despite having small spatial dimensions, these kernels extend across the entire depth of the input [4].

### 3.2. Training the NNs

For any machine learning problem, training is essential to develop the NN. Providing the required quantity and quality of training data will enhance the ability of the NN. The depth of an NN required for a specific problem can be determined by considering the training data set. A small data set is likely to generate an overfitted NN when a large number of layers are used, while the same NN trained with a large data set could be generalized to a more accurate NN [9].

## 4. Proposed DNN-Based UA OFDM Receiver

**Figure 6:**
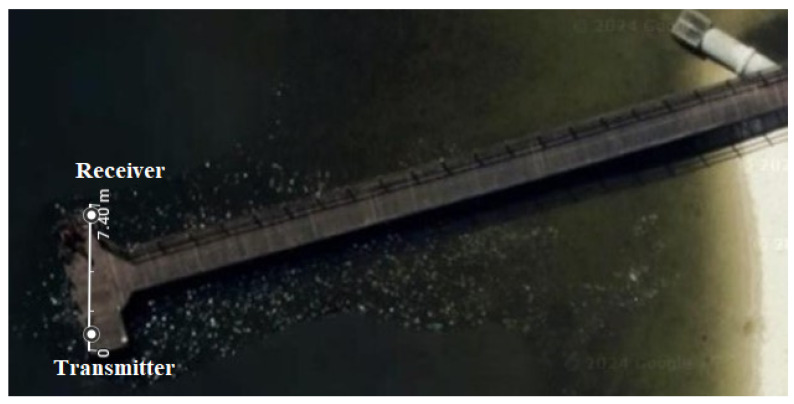
Shelly Jetty, Western Australia.

To explore UA communication using a DNN, experiments were performed in the Canning River, Western Australia. The weather was sunny during both trials and the river was at low tide for the first and high tide for the second experiment. The transducer as transmitter and hydrophone as receiver were placed diagonally across the jetty around 7 m apart as shown in Figure 6.

### 4.1. Transmitter

**Figure 7:**
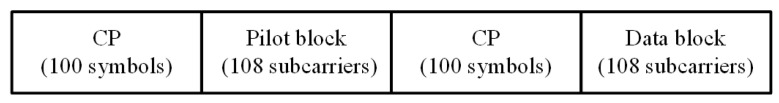
Frame structure.

**Figure 8:**
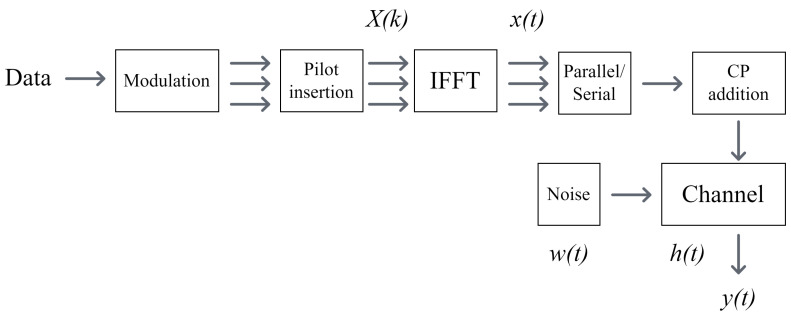
Block diagram of the transmitter.

Each transmitted frame contains one OFDM data block and one pilot block with $N_{p}=N_{c}=128$ subcarriers in each block as shown in Figure 7. The data and pilot symbols are modulated by the QPSK constellations. Hence, one symbol is encoded by two bits. The pilot block has null subcarriers in every sixth position. Then, 10 subcarriers are removed from both ends of $N_{p}=N_{c}=128$ to avoid spectral leakage in the frequency band. IFFT is applied to each OFDM symbol to convert it into the time domain signal x(t). The parallel data are converted to serial and a CP with the length $T_{cp}=100$ longer than the channel delay spread is added to the time domain symbol as illustrated in Figure 8. This data set is converted to a waveform and transmitted through a transducer into the underwater channel h(t).

### 4.2. Receiver

**Figure 9:**
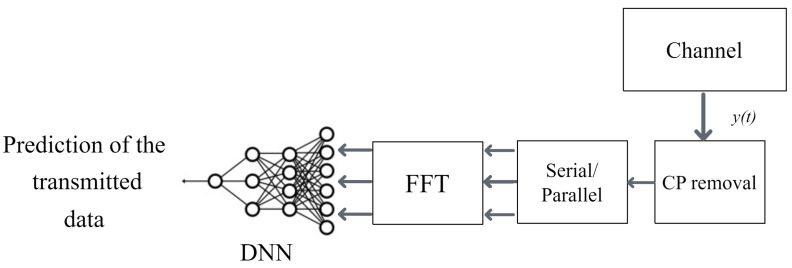
Block diagram of the receiver.

As shown in Figure 9, the signals received by the hydrophone are downshifted and the CP is removed. The fast Fourier transform (FFT) is used to convert the signal into the frequency domain after transferring the serial data into parallel. We randomly chose 10 data and pilot subcarriers from the 108 data and pilot subcarriers to reduce the training time. Then, the data frame is fed into the regression-based NN which predicts the transmitted data directly from y(t) (1) eliminating channel estimation, equalization, and demodulation as shown in Figure 9. The performance of the receiver greatly depends on the training of the network. The neural network is trained using the data gathered from the river, both transmitted and received. The deep neural network analyzes the channel characteristics and Doppler effects of the received signal, adjusting its internal settings accordingly. The weights and biases of each layer of the DNN are determined using the back-propagation algorithm with a stochastic gradient descent and a fixed step length. The loss function for regression problems is the mean-squared error *L*, as defined in Equation (10), which measures the discrepancy between the received data and the predictions made by the DNN.

(10) $$L=\frac{1}{N}\sum_{k=0}^{N-1}{\left(\hat{b}\left(k\right)-b\left(k\right)\right)}^{2}$$

where *N* is the number of bits, and $\hat{b}\left(k\right)$ and b(k) are the predicted bit and the ground truth, respectively [10].

### 4.3. Proposed DNN Architecture

**Figure 10:**
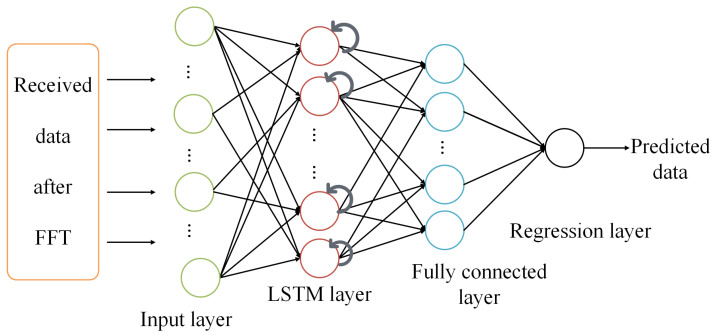
Architecture of the LSTM-based receiver.

In this experiment, three regression-based networks, an MLP, an LSTM, and a CNN, are utilized for performance comparison. This comparison provides insight into the behavior of these networks in a unique scenario. In the proposed design, the MLP has a total of four layers including a sequence input layer with 40 neurons, two fully connected hidden layers with 80 and 4 neurons, respectively, and a regression output layer shown in Figure 3. As this network has simple layers and connections, it can train fairly well even with a small data set. The design of the network utilizing LSTM consists of a sequence input layer containing 40 neurons, followed by an LSTM layer with 80 neurons, a fully connected layer with 4 neurons, and a regression output layer, illustrated in Figure 10. It is basically an MLP with an LSTM layer following the input layer. The LSTM network has more complex internal connections and requires more data to tune its internal parameters. In this sequential data problem, the architecture of CNN is chosen as a multi-layered NN with a 2D input layer ([20-by-2]), a convolutional layer containing eight filters measuring 4-by-1 and a fully connected layer with four neurons, followed by a regression output layer, as in Figure 5.

### 4.4. Training the Proposed NNs

In supervised learning, training data for the NN are created using both the transmitted and received data, as shown in Figure 3. The NN learns how transmitted and received data relate to channel characteristics from the training data set and adjusts its internal parameters accordingly. The training data set consists of both transmitted and received data. In trial 1 and trial 2, 2000 and 4000 packets of OFDM frames, respectively, are transmitted and received through a UA channel. The symbols in the pilot frame are randomly generated and, once generated, they are fixed for all packets. A data frame is randomly generated for each packet. These fixed pilots are used to learn the features of the training data set. We selected 10 subcarriers randomly from the 108 pilot and data subcarriers in each packet to form the received data frame. Typically, training a network with a large amount of data, a large number of layers, and numerous neurons can yield better performance, but it requires a long training time. When this trained network is used in a communication system with a time-varying channel, it may not provide accurate predictions because the newly input data differ significantly from the training data set. Due to the time-varying nature of the channel, the DNN must adapt to the newly input data, necessitating retraining of the original parameters. A smaller number of parameters reduces training time and is more suitable for real-time implementation. To achieve this, a smaller training data set is required. By selecting just 10 data subcarriers and 10 pilot subcarriers from a total of 108 subcarriers for feature extraction in the training data generation phase, we can reduce the network’s parameters and speed up the training process. The NN will predict the transmitted data when the testing data set with same number of subcarriers of identical indexes is provided to the trained network.

Fifty percent of the receiver’s recorded data is utilized for training and validation. The second half is utilized as test data for the final assessment. Training data are created by choosing odd-numbered packets of the received data, which provides the NN with complete channel information, while even-numbered packets are reserved for testing purposes. The transmitted data are used as labels for the received data in the training data set as shown in Figure 3. In the training data set (half of the received data), 80% is used for training and 20% is for validation. From these training data, the NN will learn the channel information and the relationship between the transmitted and received data.

## 5. Experiment Setup

**Figure 11:**
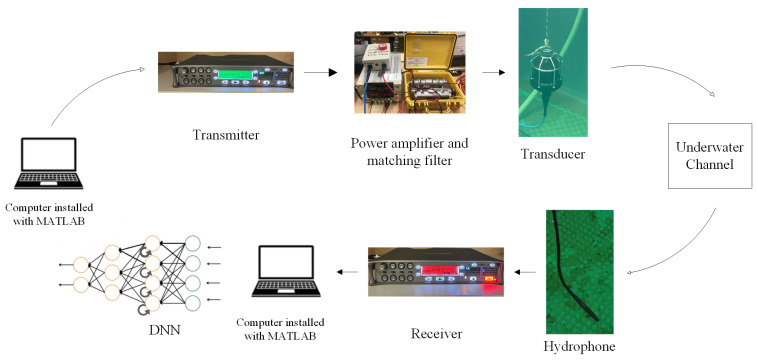
River trial setup.

To explore the UA communication using DNN, experiments were performed in the Shelley Jetty, Canning River, Western Australia. It was the summer time and the river was at low tide for the first and high tide for the second trial. The transmitter and receiver were placed diagonally across the jetty as shown in Figure 6 around 7 m apart. The receiver was positioned at 1.5 m and 2 m in these two trials, respectively, to assess the performance of DNN receivers across different channel profiles at different depths. Both river trials were performed on the receivers with the same DNN architectures. The experiment setup is shown in Figure 11. Note that the transducer and hydrophone photos were taken during a tank trial. The waveform generated from the data set described in Section 4.1 was transferred to the transmitter (SOUND DEVICES 788T). This device is capable of both playing and recording the sound signals. Therefore, we could use this to control the gain of both the transmitted and received signals. The SOUND DEVICES 788T system was connected to a power amplifier (JVC KS-AX3300) and an impedance matching network (designed and built in house). The amplified signal was transmitted to the channel through a transducer (Chelsea Technologies CTG0052). The transducer used in this experiment operates within a frequency range between 10 and 14 kHz, with a transmit sensitivity of 132 dB re 1 μPa/V at 1 m. The signals captured by the hydrophone (Reson TC 4034) were recorded using a SOUND DEVICES 788T receiver. The hydrophone was omnidirectional horizontally, covering a broad frequency range from 1 Hz to 480 kHz, and had a receiving sensitivity of −218 dB re 1 V/μPa at 1 m. The data were fed to the NN after producing the training and testing data sets from the received and transmitted data. Training data generation involved using OFDM packets with 10 data subcarriers and 10 pilot subcarriers from a total of 108 subcarriers. During these tests, a total of 4 bits were recovered from each OFDM packet.

## 6. Performance Results

The performance results of the proposed receiver design are explained in detail in this section. Section 6.1 presents the simulation results, Section 6.2 depicts the results from an indoor water tank test and Section 6.3 explains the results of the two river trials in detail. The performance of the proposed NN-based receivers is evaluated by changing the epoch rate, number of layers, and size of the training and testing data sets. The other parameters of the NNs are selected as follows: the optimizer algorithm is chosen as adam, the initial learning rate and learning rate drop factor are 0.01 and 0.1, respectively, and the mini-batch size is 100.

### 6.1. Simulation Result

**Figure 12:**
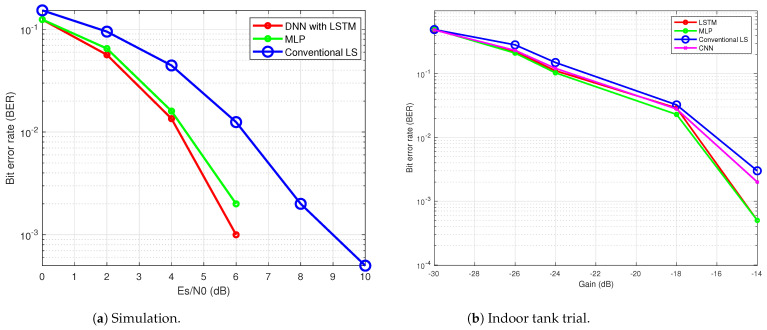
BER performance of the proposed NN-based receiver.

The performance of the proposed DNN-based receiver was previously evaluated through simulation in [34], where the channel was modeled with 15 paths and a 20-sample delay spread. As illustrated in Figure 12a, the BER performances of the DNN with an LSTM layer, the conventional LS method, and the MLP are compared. The results demonstrate that the DNN-based receivers outperform the LS-based receiver, since the DNN-based receivers are able to learn complex non-linear relationships and adjust their internal parameters, often resulting in a superior performance compared to the conventional LS method.

### 6.2. Indoor Tank Results

**Figure 13:**
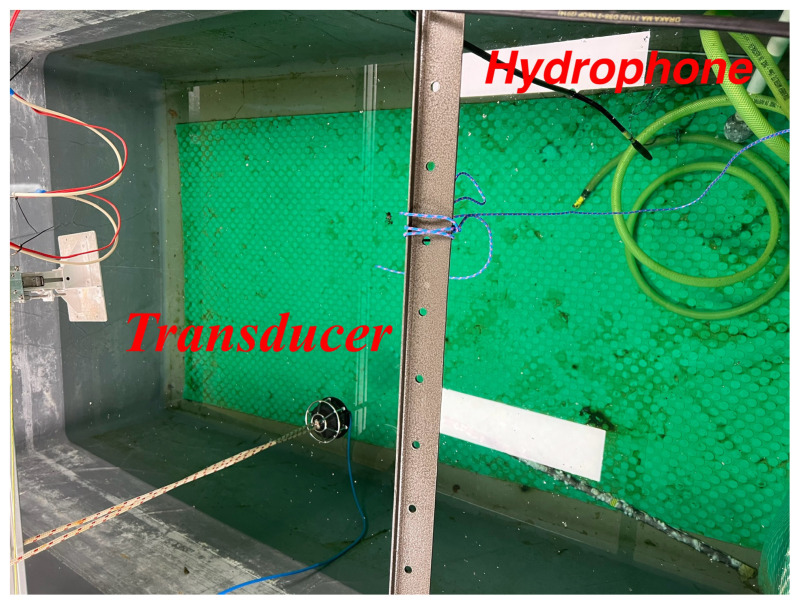
Tank setup.

**Figure 14:**
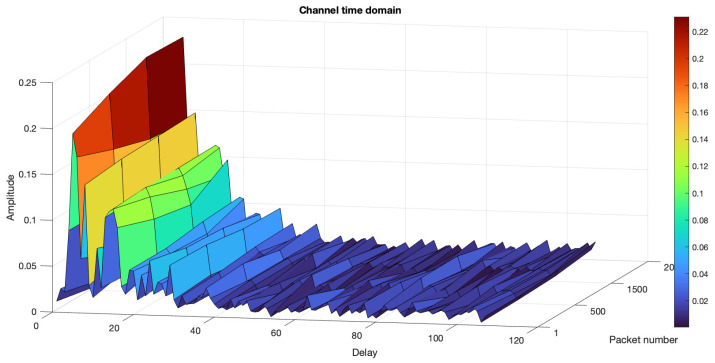
Indoor tank channel profile.

The proposed NN-based receiver was also tested in an indoor tank trial, as detailed in [35]. The water tank, shown in Figure 13, has the dimensions of 2.5 m in length, 1.5 m in width, and 1.8 m in depth, with the channel profile illustrated in Figure 14. In the experiment, the transmitter and receiver were placed 3 m apart. The results depicted in Figure 12b demonstrate that the MLP and LSTM models outperformed both the CNN and the conventional receiver using the LS channel estimation method. The superior performance of the NN-based receivers, LSTM, MLP, and CNN, compared to the conventional LS method, is attributed to their ability to compensate for non-linearity in UA channels (2). However, the CNN performed less effectively than the other two NN models, due to the limited data available to sufficiently tune its complex parameters.

### 6.3. River Trial Results

The performance results from both simulation and tank evaluations motivated the trials conducted in the Shelley Jetty, Canning River, Western Australia on the proposed NN-based receiver.

#### 6.3.1. River Trial 1

**Figure 15:**
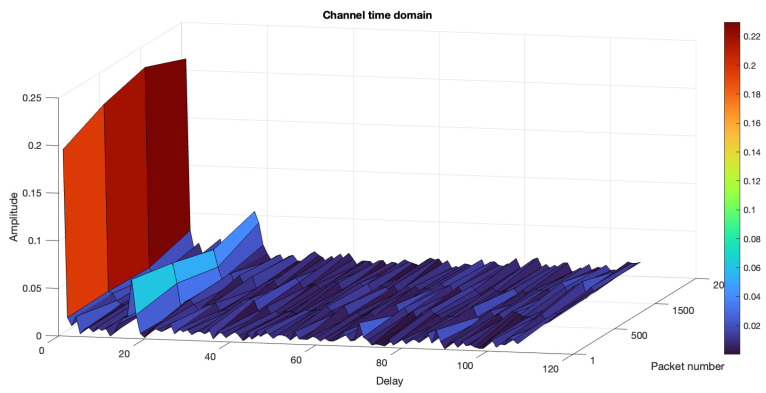
River trial 1 channel profile.

**Figure 16:**
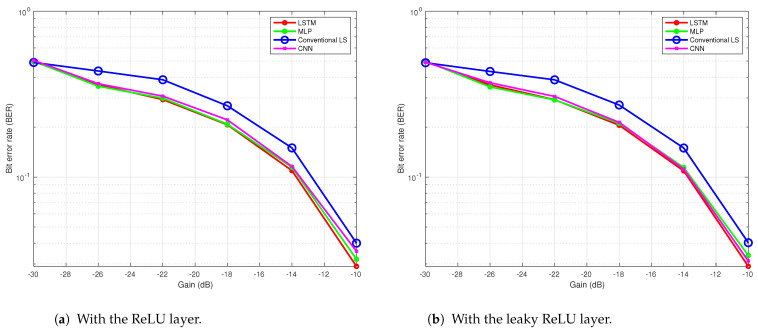
BER performance of the NN-based receivers in river trial 1.

The first river trial was conducted on a sunny day on 7 March 2024 at low tide in the river. Both the transmitter and the receiver were placed 1.5 m deep underwater. The channel profile of the river during this time is shown in Figure 15. During the first trial, we sent 2000 OFDM frames (equivalent to 4.38 minutes) six times using transmitter gains of −10 dB, −14 dB, −18 dB, −22 dB, −26 dB, and −30 dB. Changing the transmitter gain resulted in variations in the SNR received by the receiver. The MLP, LSTM, and CNN receivers had the same architecture as used for both the simulation and tank trials, which is illustrated in Section 4.3. The performance of the NN-based receiver in this trial with a ReLU layer for MLP, LSTM, and CNN is illustrated in Figure 16a and with a leaky ReLU in Figure 16b. In both the plots, the NN-based receivers performed better than the conventional LS method. Replacing ReLU with leaky ReLU improved the performance of CNN in Figure 16a. In both cases, LSTM performed better than all the other methods.

#### 6.3.2. River Trial 2

**Figure 17:**
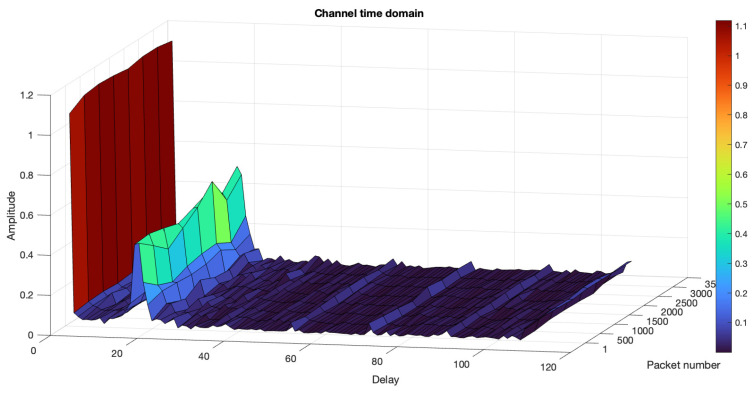
River trial 2 channel profile.

**Figure 18:**
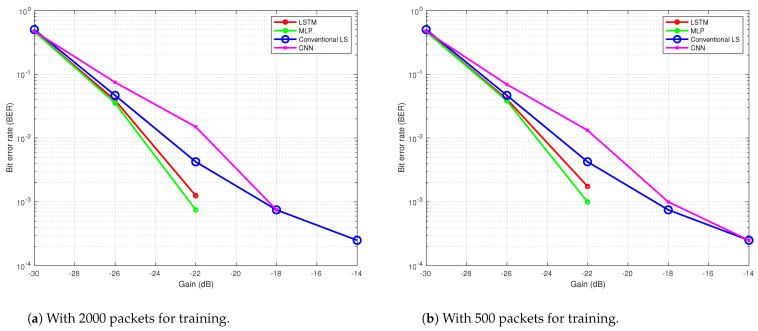
BER performance of the NN-based receivers in river trial 2 with 1000 packets for testing, 4 layers and 200 epochs.

During the second trial on 27 March 2024, the tide was high and breaking waves created plenty of impulsive noise. The transmitter and receiver were placed 2 m deep in the river. We transmitted 4000 OFDM frames (9.16 min) in the same procedure as the first trial. The channel profile of the river during the second trial is shown in Figure 17. The performances of the three NN-based receivers and the conventional method are evaluated and compared. Figure 18a show the results obtained from this trial after training with 2000 packets and testing with 1000 packets of data with ReLU layer. The MLP and LSTM architectures performed better than the conventional LS method and CNN. In Figure 18b, only 500 packets are used to train the NN but 1000 packets are used to test it. This result proves that fewer training data will reduce the performance of the NN-based receivers.

**Figure 19:**
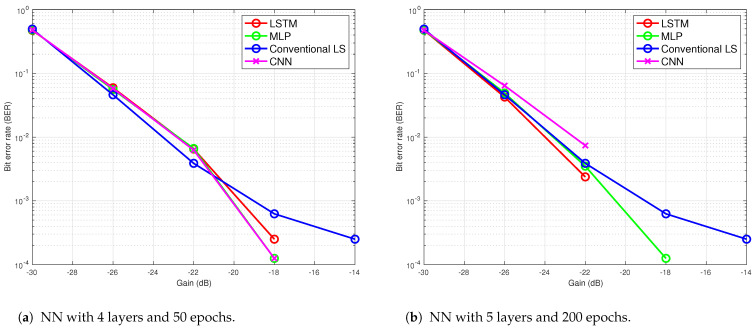
BER performance of the NN-based receivers with 2000 packets for training, 2000 packets for testing in river trial 2.

Figure 19a shows the performance of the four methods with fewer training epochs. A reduced number of epochs increased the BER in all three NN architectures and reduced the performance compared to the conventional method. The same NN architectures used in trial 1 are followed in Figure 18a and Figure 19a. To determine the effect of an increase in the number of layers on the performance, five layers are used in all NNs by adding a fully connected layer as the third layer with 50 neurons in the next plot. The result is shown in Figure 19b, where 200 epochs are used. Adding more layers to the architecture reduced their performances. These results show that the trial and error method provides suitable architecture and parameter numbers for individual problems. Table 1 presents a comparative analysis of the performance metrics for the three networks against the traditional LS method.

## 7. Conclusions

UA communication using DNN was tested by conducting multiple trials in the Canning River, Western Australia. The results obtained from the trials proved that the DNN-based receiver performed better than the conventional receiver in all the experiments. The same DNN architecture-based receiver was tested in simulation and indoor tank trials in the past also proving the same. We trained and tested the networks in various scenarios and concluded that the MLP and LSTM networks performed better than CNN and the conventional method in almost all the experiments conducted. By conducting further study on the CNN architecture modification, its performance could improve. NNs have the ability to learn channel information, and the relationship between the transmitted and received data from the training data, and compensate for the non-linearity in the channel. Therefore, NN-based receivers have more possibilities in UA communication systems, compared to the conventional method. More experiments need to be conducted to investigate the performance of a DNN-based receiver in a moving receiver or transmitter scenario.

## Figures and Tables

**Table 1:** Performance evaluation of the three networks compared to the conventional LS method (numbers represent the performance rankings).

Network	Layers	Neurons	Performance Evaluation (For 200 Epochs)			
			Simulation	Tank Trial	River Trial 1	River Trial 2
MLP	Sequence input layer Fully connected layer ReLU Fully connected layer Regression layer	40 80 4	2	1	2	1
LSTM	Sequence input layer LSTM layer Fully connected layer Regression layer	40 80 4	1	2	1	2
CNN	Image input layer Convolution layer ReLU Fully connected layer Regression layer	20 × 2 × 1 20 × 2 × 8 4	Not evaluated	3	3	Worse than conventional LS method

## Data Availability

Data is available upon request.
